# One-Stage Femoral Osteotomy and Computer-Assisted Navigation Total Knee Arthroplasty for Osteoarthritis in a Patient with Femoral Subtrochanteric Fracture Malunion

**DOI:** 10.1155/2014/645927

**Published:** 2014-09-08

**Authors:** C. H. Jason Fan

**Affiliations:** Department of Orthopaedics & Traumatology, Alice Ho Miu Ling Nethersole Hospital, 11 Chuen On Road, Tai Po, New Territories, Hong Kong

## Abstract

Extra-articular femoral deformity in total knee arthroplasty (TKA) is realigned by either intra-articular correction or extra-articular osteotomy. The more distant the deformity is away from knee joint, the more likely it is corrected by the former method. No report described the use of antegrade cephalomedullary femoral nail to fix the osteotomy followed by computer-assisted navigation TKA. This report described the unusual use of this method to manage a 64-year-old man with femoral subtrochanteric fracture malunion and osteoarthritis of knee. He demonstrated a satisfactory functional outcome and good lower limb alignment.

## 1. Introduction

Mechanical axis deviation of the lower limb and malorientation of the knee joint due to posttraumatic angular deformity of femur can cause osteoarthritis of knee. To perform total knee arthroplasty (TKA) with proper restoration of mechanical axis of lower limb, this can be achieved by either intra-articular or extra-articular correction [1, 2]. To avoid the problem of complex ligament balancing, one-stage or two-stage femoral osteotomy and TKA can be adopted. Corrective osteotomy can be fixed by a number of methods including plating, retrograde intramedullary nail, and press-fit long-stemmed femoral component while TKAs were done using the intramedullary or extramedullary guide [3, 4]. In this report, one-stage femoral osteotomy fixed by antegrade cephalomedullary nail followed by TKA done under computer-assisted navigation was done to achieve a satisfactory mechanical axis of lower limb and well-balanced knee.

## 2. Case Report

A 64-year-old man sustained right thigh and knee injuries when he had a road traffic accident in 1982. Right knee operation was performed to fix the proximal tibial fracture. He was then put on skeletal traction in a hospital for a few months and recovered gradually. Afterwards, he noticed right thigh deformity and right lower limb shortening. He was otherwise asymptomatic and resumed his duty as a manual worker.

He had his first orthopaedic consultation in 2009 and complained of gradual onset of right knee pain for 4 years. He could manage the knee pain with soft knee brace and analgesic initially. In 2012, his right knee pain worsened and he could walk unaided for 20 minutes only. He needed banister assistance when going upstairs and downstairs.

Physical examination showed mild right genu varum of about 10 degrees. There were one long medial parapatellar scar and one short anterolateral proximal tibial scar. Medial joint line was tender. Right knee active range of motion was from zero to 100 degrees. Posterior sagging, anterior and posterior drawer tests, and varus and valgus stress tests were all negative. Both tibial segments were equally long. His right femur was one centimeter shorter at the level below the greater trochanter. Palpable deformity was noted at the anterolateral aspect of proximal third of his right femur. There was no flexion contracture of the right hip. Right hip could be flexed to 60 degrees. Right hip abduction range was 20 degrees which was about half of the left hip abduction. Right femoral neck anteversion was about 20 degrees which was similar to the left hip.

**Figure 1:**
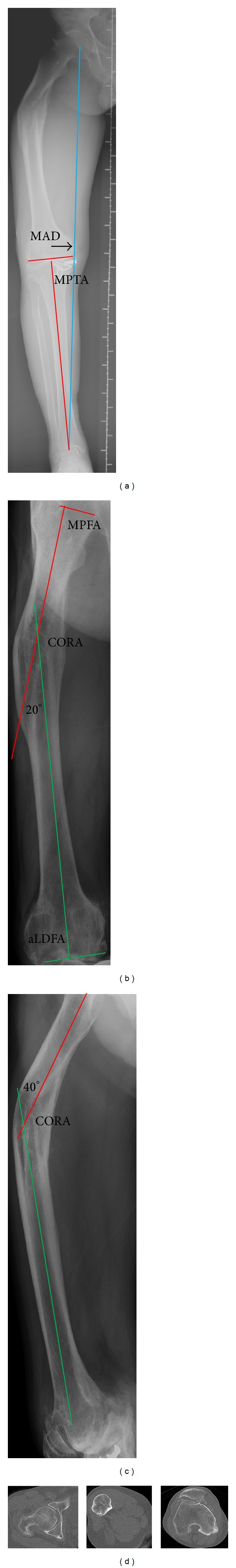
(a) Standing scanogram of the right lower limb. Mechanical axis deviation (MAD) is 5 cm from knee center. Medial proximal tibial angle (MTPA) is 84°. (b) Anteroposterior X-ray of right femur. Medial proximal femoral angle (MPFA) is 89°. Anatomical lateral distal femoral angle (aLDFA) is 89°. Lateral angulation at the center of rotation of angulation (CORA) is 20°. (c) Lateral X-ray of right femur. Anterior angulation at CORA is 40°. (d) Computer tomography of right hip, right femoral shaft at apex of deformity, and right femoral condyle. The femoral neck anteversion is 15°. The medullary canal is obliterated at the apex of deformity.

Standing scanogram of right lower limb (Figure 1(a)) showed right femoral subtrochanteric fracture malunion, old tibial plateau, proximal fibular shaft fracture, four metal staples in the medial tibial plateau, and tricompartmental osteoarthritis of the right knee. When the anatomical axes of proximal and distal femoral segments were drawn on the anteroposterior (AP) (Figure 1(b)) and lateral radiographs (Figure 1(c)) of the right whole femur, the center of rotation of angulation (CORA) was inside the bone and corresponded to the obvious apex of angulation. This implied that neither a translation deformity nor multiapical angular deformity was present. Computer tomography from right hip to right knee (Figure 1(d)) was performed to see any rotational deformity.

When planning the distal femoral bone cut by drawing perpendicular line to the mechanical axis of right femur in AP and lateral radiographs, the origin of lateral collateral ligament was likely jeopardized and the anterior cortex of distal femur had to be significantly notched to be put in the femoral component. Therefore, intra-articular correction of this extra-articular femoral deformity and osteoarthritis of knee would not be successful. Femoral osteotomy and TKA would be necessary. One-stage operation was performed in August 2012 in order to achieve good relief of knee pain and satisfactory lower limb alignment. The apex of deformity was at the subtrochanteric region of the right femur and long gamma 3 nail (Stryker) was chosen to fix the osteotomy at this CORA to allow immediate postoperative full weight-bearing walking. Posterior stabilized TKA (Triathlon, Stryker) was done using Stryker's OrthoMap Articular Surface Mounted (ASM) Knee Navigation because conventional intramedullary guide would not be possible.

**Figure 2:**
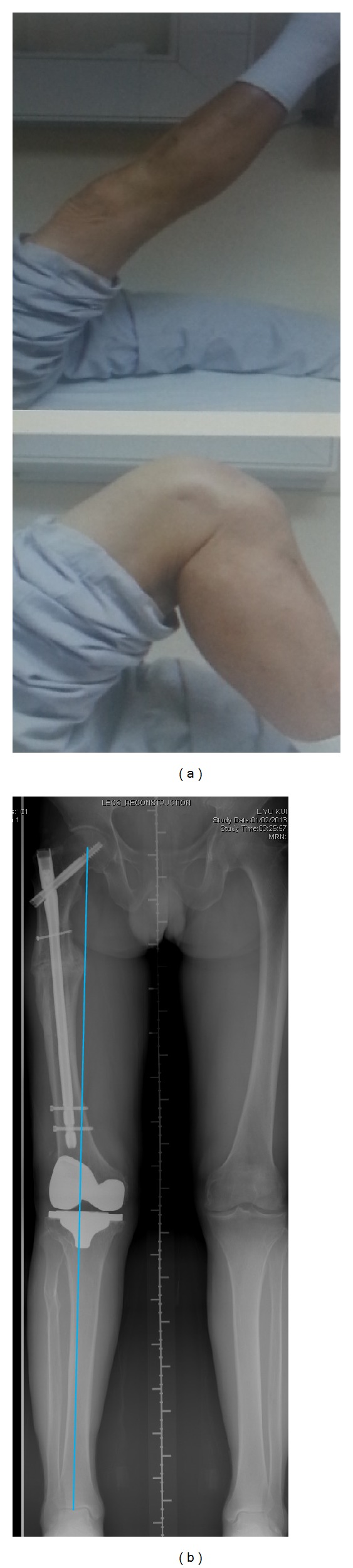
(a) Clinical photos showing active right knee range of motion. (b) Standing scanogram of both lower limbs showing satisfactory right lower limb mechanical axis and equal limb lengths.

He was last followed up in the clinic in April 2014. He had no pain at his right thigh and knee. He could walk unaided and unlimited. His active right knee range was from zero to 110 degrees (Figure 2(a)). The function score was 80 and his right knee score was 97. Standing scanogram (Figure 2(b)) showed healed femoral osteotomy and normal mechanical axis of right lower limb.

## 3. Surgical Technique

The patient was placed supine on the traction table. The CORA was located under X-ray control. Closing wedge osteotomy was done with the proximal and distal bone cuts perpendicular to the corresponding femoral segments. The obliterated medullary canal was opened with 3.2 mm drill bit. Because of the soft tissue contracture at the concavity of the deformity, manual correction by assistant alone was difficult and not sustainable. Fixator (Hoffmann II external fixation system, Stryker) assisted nailing [5] was adopted to allow better control of the deformity correction. Two 4.0 mm half pins were placed at the posterior half of the proximal segment and another two placed near the posterior cortex of the distal segment. These pins acted as blocking pins to narrow the width of the medullary canal and assisted in the deformity correction.

After the deformity was partially corrected by manual reduction and held in place by the external fixator, the entry site for the nail was located. It was important to place it slightly posterior to the anterior third of the tip of greater trochanter, so that the nail could be negotiated through the narrowed medullary canal to correct the deformity.

Long gamma 3 nail of length 340 mm was chosen to avoid too distal placement of the nail tip, which may interfere in the femoral bone cut in TKA. After the passage of nail and fixation of the lag screw and distal locking screws, the external fixator was loosened. The proximal femoral varus deformity did not recur. Poller screw [6] was not inserted to the medial side of proximal femoral segment. Before the removal of all half pins, 4.0 mm cannulated screw (Synthes) was inserted posterior to the nail at the proximal femoral segment to act as Poller screw.

Traction was then taken off and the patient was redraped to perform the TKA. Because the old scars were present for 30 years and it was difficult to incorporate the very lateral scar in the incision, longitudinal midline incision and medial parapatellar approach was used. All the metal staples were removed and the TKA was completed under ASM computer navigation. The sterile tourniquet was removed to reopen the wound for osteotomy. Cancellous bone graft taken from the bone chips after TKA was placed around the osteotomy.

## 4. Discussion

The long-term success of TKA is dependent, in part, on proper restoration of the mechanical axis and soft tissue balancing [2, 3]. In the presence of extra-articular deformity of the femur associated with osteoarthritis of knee, this can be difficult to achieve [1–4, 7]. J.-W. Wang and C.-J. Wang [8] advocated the use of intra-articular bone resection in conjunction with TKA when the extra-articular varus deformity of femur was less than 20 degrees and the insertion of collateral ligaments was not jeopardized. However, because femoral compensatory wedge resection produces instability only in extension, femoral deformity is more difficult to correct intra-articularly [2]. Wolff et al. [2] also pointed out that feasibility of joint line resection and soft tissue balancing was determined by the degree of deformity and its distance from the knee. The larger the deformity was and the closer it was to the knee joint, the greater its impact on the knee was and the less feasible this method was. Restoration of the normal mechanical axis with intra-articular resection in the presence of notable femoral deformity might normalize the orientation of the knee, but hip adduction or abduction was still necessary to keep the knee and ankle parallel to the ground in the stance phase of gait. This might result in localized areas of stress concentration in the hip and ultimately contributed to accelerated osteoarthritis [2].

The indication of intra-articular bone resection in femoral sagittal plane deformity was less well defined. J.-W. Wang and C.-J. Wang [8] successfully managed seven patients with femoral deformity in sagittal plane (up to 25°). It was not specified whether they were flexion or extension deformity. Kuo et al. [9] reported the use of computer-assisted navigation in TKA for a 70-year-old woman with severe posttraumatic femoral deformity. The distal femoral segment was translated posteriorly and extended. The coronal deformity was varus 5 degrees. The femoral component was satisfactorily positioned and compensatory flexed to correct the extension femoral deformity without anterior notching of the femur.

Femoral osteotomy prior to TKA is preferred when there is complex triplane extra-articular deformity [7]. Two-stage procedure is indicated when the surgeon has limited experience of complex knee deformity arthroplasty. This should also be considered in young patient, as the correction of the malalignment may provide enough improvement in symptoms to delay the time to TKA. One-stage procedure is a technically difficult but effective treatment [3]. Lonner et al. [3] recommended the use of plate or locked intramedullary nail to secure the femoral osteotomy site. One of the two patients who were treated with press-fit long-stemmed femoral component had nonunion of femoral osteotomy.

In this case report, the coronal plane deformity was varus 20° in the proximal third of femur. Intra-articular bone resection in TKA was unexpectedly found to jeopardize the origin of lateral collateral ligament. In contrast to the case reported by Kuo et al. [9], this patient had flexion deformity of 40° and compensatory femoral component extension could not be adopted because of the risk of notching. Therefore, femoral osteotomy followed by TKA was unusually chosen for this proximal femur deformity. As the gentleman was 64 years old, one-stage procedure was preferable to realign the lower limb before TKA was performed. This could hasten the rehabilitation process and recovery.
